# Selective effects of a brain tumor on the metric representation of the hand: a pre- versus post-surgery comparison

**DOI:** 10.1007/s00221-022-06475-8

**Published:** 2022-10-19

**Authors:** Laura Mora, Giorgia Committeri, Marco Ciavarro, Gianna Cocchini

**Affiliations:** 1 Psychology Department, Goldsmiths University of London, London, SE14 6NW UK; 2 Institute of Advanced Biomedical Technologies, University “G. d’Annunzio”, Chieti-Pescara, Italy; 3 IRCCS Neuromed, Pozzilli, Italy

**Keywords:** Body representation, Brain tumor, Hand size, Metric representation, Sensorimotor areas, Localization task

## Abstract

Body representation disorders are complex, varied, striking, and very disabling in most cases. Deficits of body representation have been described after lesions to multimodal and sensorimotor cortical areas. A few studies have reported the effects of tumors on the representation of the body, but little is known about the changes after tumor resection. Moreover, the impact of brain lesions on the hand size representation has been investigated in few clinical cases. Hands are of special importance, as no other body part has the ability for movement and interaction with the environment that the hands have, and we use them for a multitude of daily activities. Studies with clinical population can add further knowledge into the way hands are represented. Here, we report a single case study of a patient (AM) who was an expert bodybuilder and underwent a surgery to remove a glioblastoma in the left posterior prefrontal and precentral cortex at the level of the hand’s motor region. Pre- (20 days) and post- (4 months) surgery assessment did not show any motor or cognitive impairments. A hand localization task was used, before and after surgery (12 months), to measure possible changes of the metric representation of his right hand. Results showed a post-surgery modulation of the typically distorted hand representation, with an overall accuracy improvement, especially on width dimension. These findings support the direct involvement of sensorimotor areas in the implicit representation of the body size and its relevance on defining specific size representation dimensions.

## Introduction

Body representation disorders have quickly become the focus of numerous studies as they provide the perfect opportunity to identify a network of cortical areas that play a central role in representing the body (Berlucchi and Aglioti 2010; Di Vita et al. 2016; Magnani and Sedda 2016). For instance, brain damage to the insula and premotor cortex can cause an alteration of the visuospatial or structural component of the body parts due to a failed integration of sensory motor information with other body representations (Di Vita et al. 2015). Further, damage to the right supplementary motor cortex caused a patient to experience supernumerary phantom limb (Hari et al. 1998; McGonigle et al. 2002), whereas a pervasive feeling of having four legs has been described after the removal of a right parietal meningioma (Vuilleumier et al. 1997). More recently, supernumerary finger phenomenon during drawing (i.e., drawing more than five fingers) has been described in a group of patients who underwent resection of tumors in the right anterior frontal insula and operculum (Niki et al. 2014). A recent case study presented a patient with a right temporoparietal tumor that caused complete bilateral loss of a patient’s body ownership, specifically, feeling as if his body had been ‘lost’ (Smit et al. 2018). Indeed, distortions caused by brain tumors are of particular interest because of their relatively slow progression of illness (in comparison to traumatic injuries or strokes) and the possibility of comparing the effects of injury before and after tumor resection. However, pre- and post-surgery observations on body representation disorders have been rarely reported due to the scarcity of these cases. These observations are of interest to understand the involvement of specific cortical areas in the representation of the body, the potential effects of the lesion, and the recovery of functions post-surgery.

One specific body part that has been widely studied in body representation research is the hand. Hands are used for a multitude of daily activities, such as manipulating objects, tactile perception, gestural expression, and showing affection. No other body part has the ability for movement and interaction with the environment that the hands have, and their relevance has made them the focus of numerous studies. Indeed, systematic assessments of different aspects of the size and shape of the hands have been published throughout the recent years. A large bulk of the recent research on the topic has involved a research group led by Matthew Longo that has predominantly focused on the study of the distortions of the representation of the hands. Following the study of touch anisotropies (i.e., Weber’s illusion), Longo and colleagues (2010) proposed the existence of a body model, a stored implicit mental metric representation of the body that includes information about the size of body segments (Longo et al. 2010; Longo and Haggard 2010; Serino and Haggard 2010). This body model has been assessed by different variants of the localization task (e.g., Longo 2022; Longo and Haggard 2010)*.* Briefly, participants place their hand under an occluded board to locate single landmarks on it (i.e., knuckles and fingertips), by pointing with a baton. The goal is to point to the location where participants feel their landmarks to be, relying on their *position sense* that indicates where the body is in space at any given time (Longo 2015a). By measuring the distances between pairs of landmarks (e.g., tip of the thumb and the knuckle), these authors compared the real and perceived size judgments. Consistent replications of the results have shown that, in the healthy population, fingers are underestimated in length, the hand is overestimated in width, and there is a radial–ulnar gradient in the underestimation of finger lengths (i.e., the thumb is the least distorted, while the little finger is the most) (Longo 2015a, 2022). Longo and Haggard (2011) postulated that this shared implicit representation of the body size and shape (the body model) preserves the characteristics of the somatosensory homunculus, and it is discerned both by touch and position sense (Coelho and Gonzalez 2018; Longo 2014). In their model, distances between two touches will be calculated by ‘counting’ the number of ‘pixels’ (receptive fields) in between them. This explains why an identical object touching two skin surfaces with different receptive field distributions (e.g., forehead versus the dorsum of the hand) will be perceived as larger or smaller. The receptive field geometry then informs the body model, producing the perceptual distortions in hand size representation (Longo 2017, 2022; Longo and Haggard 2011).

Despite the interest in using this task in the healthy population and the relevance of these findings, the localization task has been rarely used in the clinical population. For the first time, Longo and Haggard (2012a, b) adopted the localization task for the hand in a study of a patient with congenital limb loss, in which they found the hand size representation to be preserved even in the absence of the limb. A similar localization task was used to study the representation of lower limbs in patients with body integrity identity disorder, which again did not show any impairment in their capacity to represent their lower limbs, even though they perceived them as foreign (Stone et al. 2020). More recently, an adaptation of the localization task was used to study the representation of the hands in two patients with sensory loss. They found that a combination of reliance on visual information and extensive hand use reduced the size of the distortions when compared with healthy controls (Miall et al. 2021). These studies helped further understand the existence of a stored body model in the brain that appears stable despite insults to its integrity. Here, we present the case of a professional bodybuilder (AM) who had a tumor located in the left posterior prefrontal and precentral cortex at the level of the hand’s motor region. AM offered the occasion to investigate possible modulations of his contralesional (i.e., right) hand representation by tumor presence and resection. Considering the location of AM’s lesion, we aimed to explore the involvement of sensorimotor areas in hand size representation. For this, a task that taps into the body model, which is constructed and influenced by the homuncular representation (Longo and Haggard 2011), was needed. Hence, the localization task was a good alternative to measure the effects of the tumor on the metric representation of the hand. This task taps into the more implicit components of body representation (body model), in contrast to visual estimates that are more explicit and constructed by vision (Longo 2015a, 2017, 2022; Longo and Haggard 2012b). Indeed, previous research has identified differences in the body model of the hands using this task in participant experts in the use of hands (i.e., sign language interpreters; Mora et al. 2021), a practice that is associated with anatomical structural changes in the motor representation of the hand (Allen et al. 2013; Penhune et al. 2003; Sastre-Janer 1998). We expected to observe specific modulation of his contralesional (right) hand representation that would modify following tumor resection.

## Materials and methods

**Fig. 1:**
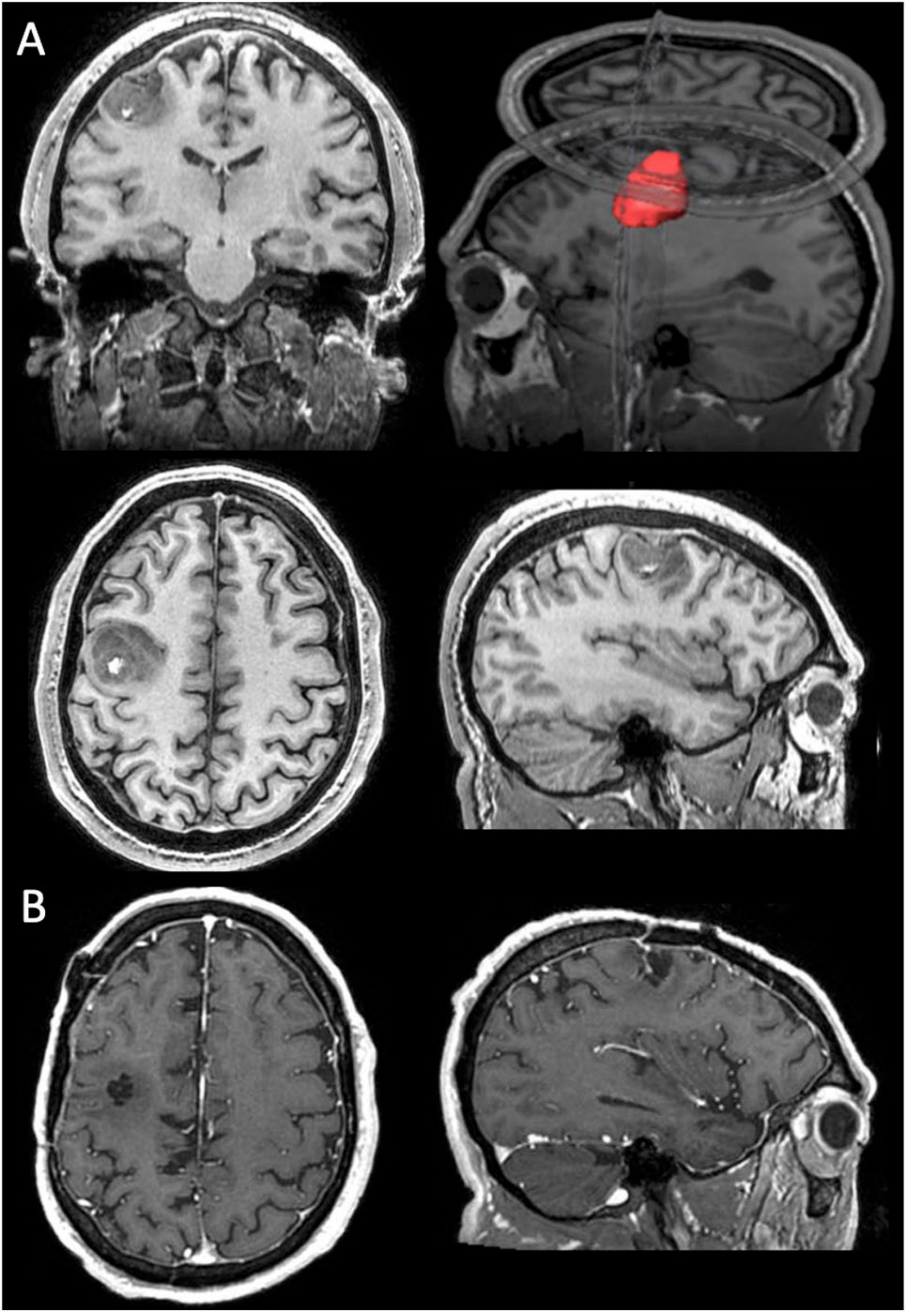
Patient AM’s T1-weighted magnetic resonance imaging scans before and after surgery. **A** T1-weighted (SPGR) magnetic resonance imaging scan in native space showing lesion location in the left posterior prefrontal and precentral cortex at the level of the hand region. Top right panel shows a 3D reconstruction of the lesion volume. Top left, bottom left and bottom right pictures represent coronal, transverse and sagittal slices centered on the lesion volume, respectively. **B** Transverse and sagittal views of the post-surgery MR scan (with T1 enhancement after gadolinium administration) in native space, coregistered to the pre-surgery scan

AM was a 41-year-old man. He had 18 years of formal education and was right-handed (laterality index = 0.91), as assessed by the Edinburgh Handedness Inventory (Oldfield 1971). He was a financial advisor and expert bodybuilder for the last 8 years. He was admitted to the IRCCS Neuromed, Mediterranean Neurological Institute (Pozzilli, Italy), where he received a diagnosis of left precentral glioblastoma (grade IV). Histopathological analysis showed a mutant IDH1 and ATRX, compatible with the diagnosis of secondary glioblastoma from an astrocytic lineage, and in accordance with the long radiological and clinical history. The patient underwent structural MRI investigations with and without contrast sequences on a 3 Tesla GE scanner both before and after surgery, as well as pre-surgical functional mapping of language and motor functions. The structural scans showed a lesion of about 3 cm^3^, localized in the left posterior prefrontal and precentral cortex, within the hand motor region (see Fig. 1). AM never reported alterations of his body perception.

AM underwent a resection of the tumor after about 5 months from diagnosis. Subpial microsurgical (i.e., under surgical microscope magnification) resection under continuous and real-time neurophysiological monitoring was performed. The procedure was conducted under local anesthesia (awake surgery) with the aid of a neuronavigation system. Surface cortical stimulation mapping showed critical sites for hand and arm contralateral movements localized outside the tumor lesion in the posterior side, and a language site eliciting dysarthria in the inferior side of the cortical boundary of the tumor. The resection was stopped when normal surrounding tissue was encountered, paying more attention when motor performance worsened, especially during fine movement evaluation.

Immediately after the surgery, AM showed dysarthria and a mild strength deficit of the right upper limb, mainly distal. Both deficits resolved within 6 days on a clinical evaluation. No further cognitive or motor deficits were reported in the following months by the patient who soon came back to work.

AM was asked to complete a formal psychometric assessment to evaluate his cognitive and motor abilities before (20 days) and after (4 months) tumor resection. Pre-surgical neuropsychological examination (see results section) did not identify any motor and cognitive deficits on the processes investigated. At the same time point before surgery and after 1 year from surgery, he was also asked to complete the task to assess the mental representation of his right (contralesional) hand.

Ethical approval was obtained by the medical ethical committee of the IRCCS Neuromed. The study was conducted in accordance with the principles of the Declaration of Helsinki (1964) and its later amendments. Data were treated anonymously. The patient voluntarily and enthusiastically took part in the study, provided an informed written consent, and could withdraw from the study at any moment without providing any justification.

### Cognitive assessment

AM completed ten subtests from the Esame Neuropsicologico Breve 2—ENB2 (tr. Short Neuropsychological Examination) (Arcara et al. 2011) which assesses general cognitive functioning: digit span (verbal short-term memory), trail-making test (A and B, selective attention), memory with interference test (verbal working memory with two temporal delays: 10 s and 30 s), story recall (immediate and delayed verbal long-term memory), overlapping figures (visuo-perceptual abilities), phonemic fluency (language and cognitive flexibility), and the clock drawing test (image retrieval, reasoning, and praxis abilities).

The Frontal Assessment Battery (FAB) is a short battery to assess frontal executive functioning problems (Dubois et al. 2000). It includes six subtests: conceptualization, mental flexibility, motor programming, sensitivity to interference, inhibitory control, and environmental autonomy. An Italian version of the FAB was administered (Appollonio et al. 2005). AM completed the test only before surgery.

### Motor function assessment

Motor functions of the hand were explored in terms of finger dexterity and maximal grip strength.

#### Finger dexterity assessment

Finger dexterity was measured with the nine-hole peg test (Kellor et al. 1971; Mathiowetz et al. 1985). This test consists of a plastic board with nine holes in it (10 mm diameter, 15 mm depth) at a distance of 32 mm apart and a shallow round dish on the opposite end. It also includes a set of nine plastic pegs (7 mm diameter, 32 mm length) which are all fitted in the holes. The board was positioned on a table, aligned with the participant’s body midline oriented in such a way that the dish was on the participant’s preferred hand side. AM was seated positioned in front of the board and was asked to pick one peg at a time and put them in the shallow dish as fast as possible, only using one hand, until all pegs were removed. Standard instructions were provided (Mathiowetz et al. 1985) and AM was allowed a short practice. The procedure started with the preferred hand (right hand), followed by the non-preferred hand. AM was allowed to support the board with the hand not being evaluated in each trial. AM was timed with a stopwatch.

#### Maximal grip strength

Maximal grip strength is normally used as a functional measure of the integrity of the upper extremity that quantifies weakness (Bertrand et al. 2015; El-Sais and Mohammad 2014). AM was tested for both the upper extremities with a Jamar digital dynamometer (Sammons Preston Rolyan, Bolingbrook, USA), and results were recorded in kilograms force (kgf). AM’s grip strength was measured in a seated position following the American Society of Hand Therapists recommendations: elbow flexed at 90°, forearm in neutral position, shoulder adducted and neutrally rotated and wrist between 0° and 30° of extension (El-Sais and Mohammad 2014; Fess and Moran 1981). The patient repeated the test twice, and the average value was considered as the result. The first session was conducted with the preferred limb.

### Hand localization task

AM’s metric representation of his right hand was assessed 20 days before and 12 months after the tumor resection by means of the localization task (e.g., Longo and Haggard 2012a, b). In this task, participants are asked to point to different body parts to discern the metric representation of their body. Since the pointing task requires movements of one hand to localize specific points of the other hand, representations of the right (contralesional) hand were assessed requiring pointing of the left (ipsilesional) hand. Representation of the left hand was not assessed as this would have required to point with the contralesional, and thus potentially affected, right hand.

**Fig. 2:**
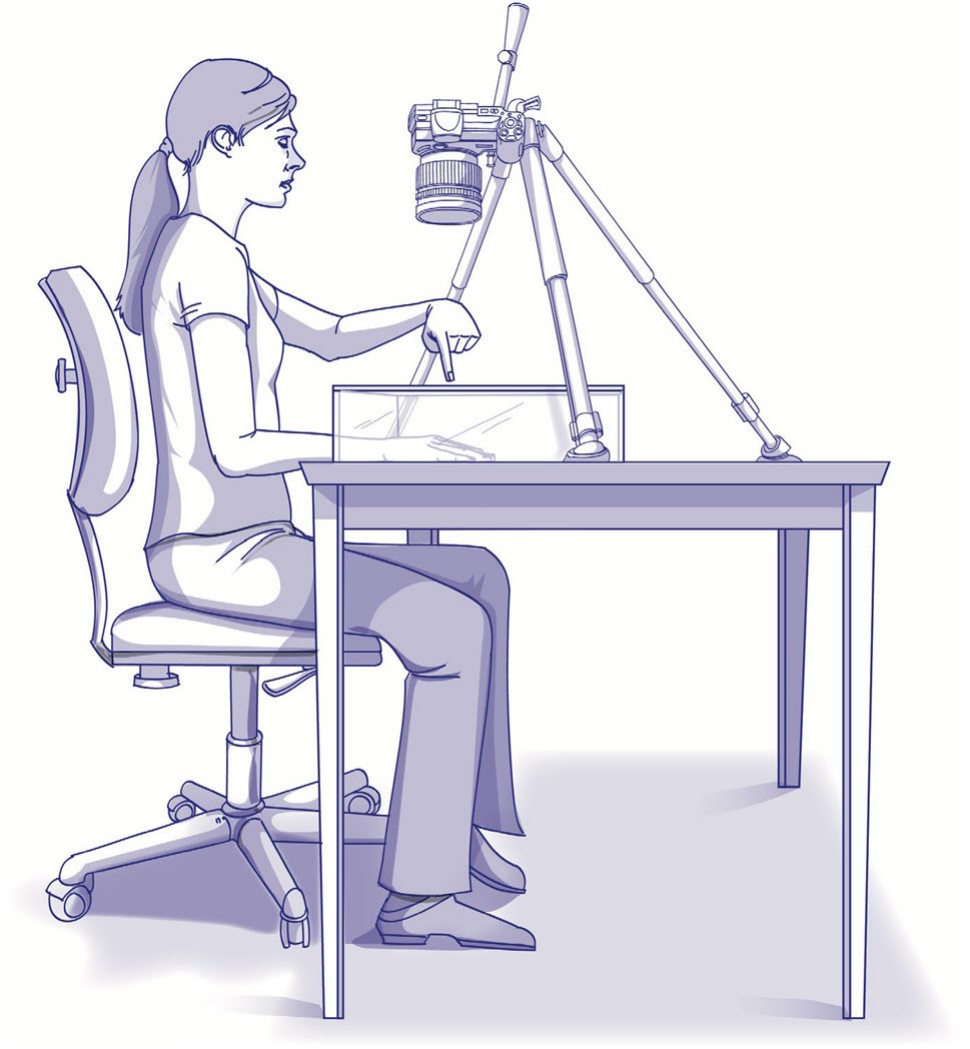
Hand localization task. Modified from Mora et al. (2021)

We used a modified version of the hand localization task, as in previous research (Mora et al. 2021). In this case, a horizontal transparent Perspex board (30 × 30 cm) was positioned on top of four metal posts (10 cm high). The board was on a table, in front of the participant. A remote-controlled camera (Nikon 6000) was placed on a tripod (90 cm height), perpendicular to the center of the board, in such a way that the camera focus was aligned with it. A small canvas (20 × 20 cm) was positioned underneath, on which AM rested the tested hand with fingers spread comfortably. AM sat in front of the table and had his eyes closed for the whole duration of the procedure. The middle finger was aligned with the participant’s body midline. They kept the hand still in this position for the whole duration of the task (Fig. 2).

AM was then asked to use his left index finger (dot drawn on the index fingernail for reference) to point on top of the board to different landmarks on the occluded right hand. There was a total of 11 landmarks requested, one at a time (5 fingertips; 4 interspaces; and the two sides of the wrist’s bones, ulna and radius). Each landmark was requested three times for a total of 33 sets of data for each assessment (i.e., pre- and post-surgery). Pointing adjustments were allowed (Kammers et al. 2009; Króliczak et al. 2006). A picture (5184 × 3456 pixels) of each pointing response was taken, and these were used to measure the accuracy of the metric representation of the hand. A measuring tape was placed on the borders of the board to provide a reference for later conversion of pixels into centimeters. To ensure understanding of the labels given to the different landmarks of the hand, AM was asked to identify these landmarks on a schematic hand picture placed in front of him during a practice trial.

#### Image processing for hand representation

The pictures were analyzed following the same method adopted in Mora et al. (2021) with a bespoke-made image analysis program using Borland C^++^ Builder (2007) that allowed conversion of pixel units into centimeters. The x and y coordinates for the real and perceived locations were obtained for each landmark (the origin was at the bottom right corner of each picture). With the coordinate data, distances (in centimeters) between pairs of landmarks were calculated, as in previous studies (e.g., Mora et al. 2021): (i) length of fingers were represented by the distance between each fingertip and adjacent interspace; (ii) the length of the hand’s dorsum was represented by the distance between the interspace between the ring and little fingers and the exterior side of the wrist; (iii) the width of the hand was represented by the distance from the interspace between the index and middle fingers, and the interspace between the ring and little fingers; and (iv) the width of the wrists was represented by the distance between the two sides of the wrists.

Further, percentages of over/underestimation for length and width perception were obtained by comparing the perceived size against the real size: [(perceived size − real size)/real size] × 100. Negative values denoted underestimation, positive values, overestimation, and zero denoted perfect performance. Percentages of over/underestimation for fingers, hand width and wrist width were averaged across the three trials to obtain a measure of the overall performance.

### Statistical analyses

Patient’s results were considered by running one-sample *t* tests to assess if the distortions were significantly different from zero. Paired-samples *t* tests were used to assess differences between assessment time (before and after surgery), where data for both sessions were available. Corrections for multiple comparisons were applied (with *p* < 0.02).

## Results

### Cognitive and motor function assessment

Scores for AM’s performance on cognitive tests, normal ranges and cutoff scores are presented in Table 1. AM showed normal performance in all tests run before and after the surgery.

**Table 1:** AM’s neuropsychological and motor assessment results

Tests		Range	Pre-surgery	Post-surgery	Cutoff
ENB2					
Digit span		0–8	7	7	5
Story recall—immediate		0–28	18	16	8
Story recall—delayed		0–28	19	19	11
Memory with interference—10 s		0–9	9	9	6
Memory with interference—30 s		0–9	8	9	4
Trail making test—A (s)		NA	32	36	55
Trail making test—B (s)		NA	91	92	142
Phonemic fluency test		0–34	12	10	10
Overlapping figures test		0–50	38	NA	32
Clock drawing test		0–10	9	9	8
FAB		0–18	18	NA	13.5
Motor assessment					
					Mean (SD)*
Nine-hole peg test (s)	RH		19.9	20.7	18.54 (2.88)
	LH		19.6	20	18.49 (2.42)
					Range*
Maximal grip strength (Kgf)	RH		42.9	41.8	35.5–55.3
	LH		37.25	37.5	35.5–55.3

ENB2. Accuracy scores (i.e., number of correctly reported elements) for each subtest, except for the trail-making test A and B for which the time of execution is provided. FAB, total score was obtained for the Frontal Assessment Battery. Cutoffs for each ENB2 subtest and for the FAB are provided with reference to the patient’s age and education level. A performance below (above for the trail-making test) the cutoff is considered pathological

*NA* not available, *RH* right hand, *LH* left hand

^*^Normative data

Concerning motor abilities (see Table 1), AM’s performance on finger dexterity assessment was compared with normative data from a recent study (Oxford Grice et al. 2003). Crawford *t* tests for single case were run for both hands, before and after surgery and results did not reach significance for any (all p > 0.05), confirming that AM performed within norms in both pre- and post-surgery sessions.

Also, AM’s maximal grip strength was compared with norms provided by the device’s manual Suaver Digital Dynamometer, 90 kg and performance was within norms for both hands and both sessions.

### Hand localization task

**Fig. 3:**
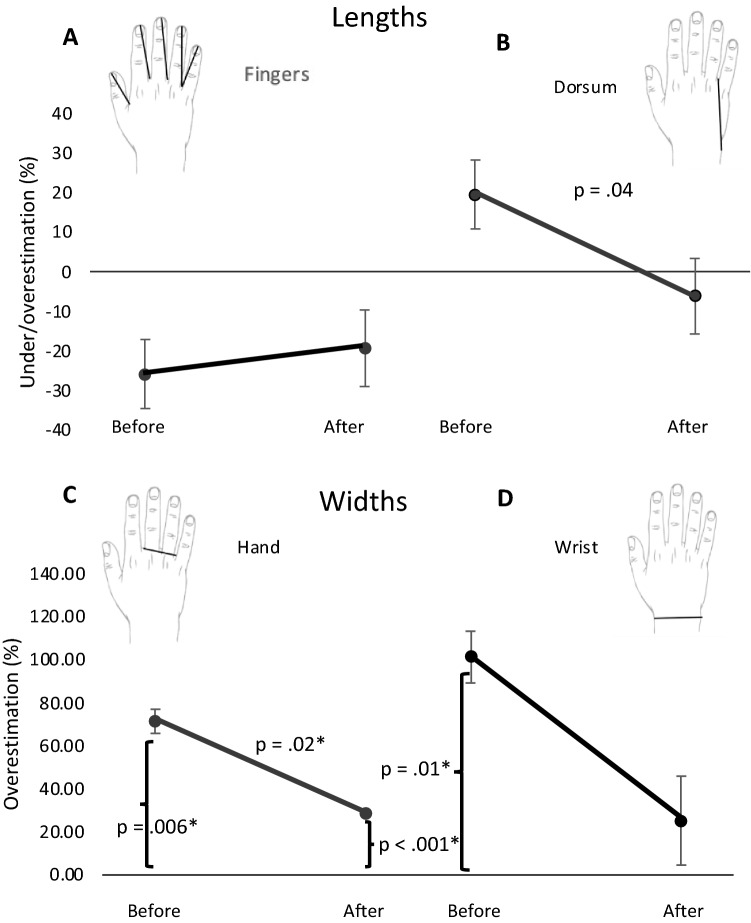
Distortion of widths and lengths. Representation of perceived underestimation and overestimation (%) of finger lengths (**A**), hand dorsum (**B**), hand width (**C**) and wrist width (**D**), for the right hand. Error bars represent the standard error of the mean. * Significant differences after correction for multiple comparisons

**Fig. 4:**
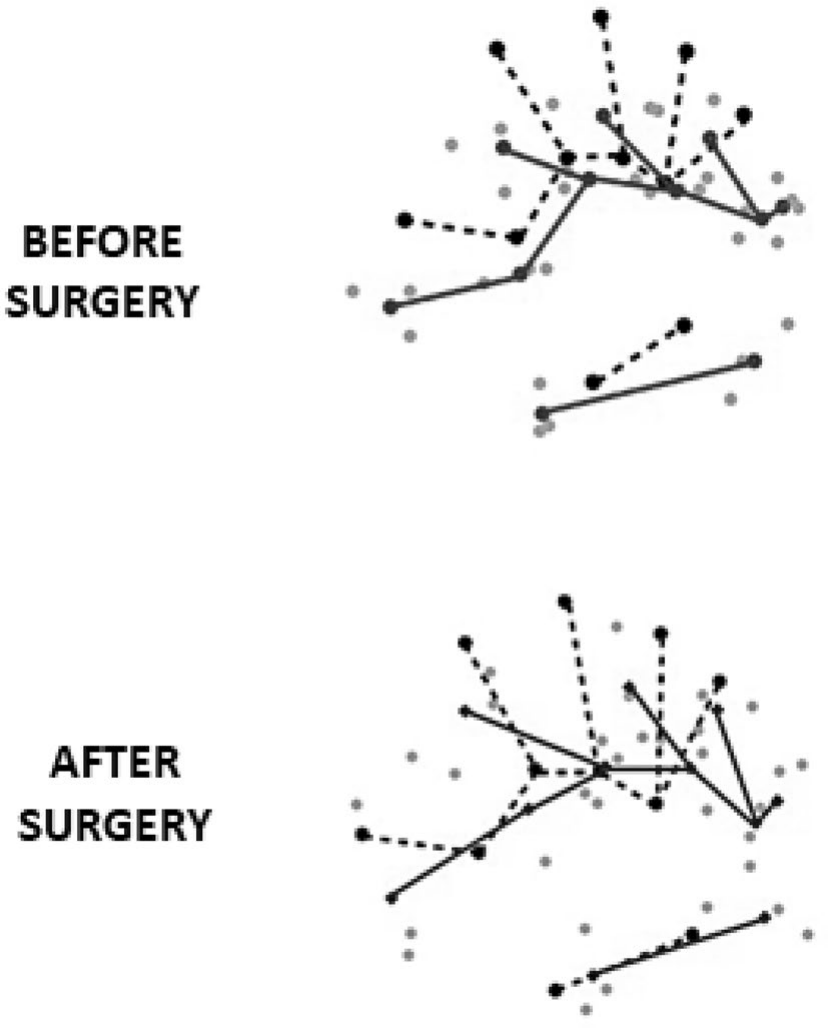
Cartographic maps for the real and perceived representation of the right hand. Black dotted lines represent the real size of the hands. The light gray dots represent all AM’s pointing responses from which averaged representation is calculated (solid lines)

The perceived size of the length of fingers, length of the dorsum of the hand, width of the hand and width of the wrist were obtained. The results are illustrated in Figs. 3 and 4.

#### Finger lengths

The perceived size of all fingers was averaged to obtain a single overall percentage of distortion. Before surgery, AM underestimated the finger lengths (*M* = − 25.79%; SD = 15.06), but the distortion did not differ significantly from zero [*t* (2) = − 2.97, *p* = 0.097, *d* = − 1.71]. Also, after the surgery, he showed an overall underestimation (*M* = − 19.23%; SD = 16.68), but not significantly different from zero [*t* (2) = − 1.99, *p* = 0.18, *d* = − 1.15] (see Fig. 3A). In line with this, significant differences before and after surgery were not found [*t* (2) = − 0.39, *p* = 0.73, *d* = − 0.23]. These results suggest that finger length representation did not change significantly after tumor resection (see Figs. 3A and 4).

#### Dorsum length

Before the surgery, AM tended to overestimate the length of his right hand dorsum (*M* = 19.55%, SD = 14.78) but the distortion was not significant [*t* (2) = 2.29, *p* = 0.15, *d* = 1.32]. After surgery, AM showed the opposite trend with a mild underestimation of the length of the dorsum (*M* = − 6.12%, SD = 11.39), which was not significantly different from zero [*t* (2) = − 0.93, *p* = 0.45, *d* = − 0.54]. Despite the opposite direction of the average error between sessions, the difference did not survive multiple comparison and only showed a trend [*t* (2) = 4.83, *p* = 0.04, *d* = 2.79]. These findings are not conclusive, but the trend between sessions leaves open a question about a possible modulation of the tumor removal on this aspect of hand representation (see Figs. 3B and 4).

#### Hand width

An overall overestimation of the right hand width was present in pre- and post-surgery (see Fig. 3C). In detail, the right hand was significantly [*t* (2) = 12.49, *p* = 0.006, *d* = 7.21] overestimated before surgery by a 71.38% (SD = 9.89). After tumor resection, the hand width was still significantly [*t* (2) = 194.2, *p* < 0.001, *d* = 112.12] overestimated, but to a lesser extent (*M* = 28.59%; SD = 0.36). Difference between the two sessions was significant [*t* (2) = 7.49, *p* = 0.02, *d* = 4.33], confirming a significant reduction of the distortion after surgery and providing evidence of an improvement on its representation (see Figs. 3C and 4).

#### Width of wrists

Lastly, before surgery AM significantly [*t* (2) = 8.57, *p* = 0.01, *d* = 4.95] overestimated the width of his right wrist (*M* = 101.22%; SD = 20.47). In contrast, the distortion after surgery appeared much reduced (*M* = 25.03%, SD = 35.81) and not significantly different from zero [*t* (2) = 1.21, *p* = 0.35, *d* = 0.7]. The difference between sessions was not significant [*t* (2) = 3.39, *p* = 0.077, *d* = 1.96]. Despite a more accurate representation of the wrist size after the surgery, these findings do not support a relevant modulation due to resection of the tumor (see Figs. 3D and 4).

## Discussion

AM’s cognitive abilities were assessed with a wide range of cognitive tests and his performance were well above cutoffs both before and after the surgery. He also performed within norms in motor tasks assessing grip and strength for both hands and no relevant difference between the two sessions emerged. These findings suggest a stable and normal performance on both cognitive abilities and motor skills.

On the hand localization task, AM showed the typical pattern of distortion for the hand representation, underestimation of length (~ 20–30%) and overestimation of width (~ 60–80%) that was first described in Longo and Haggard’s (2010, 2012a, b) studies and extensively replicated in literature (see Longo 2022 and Peviani and Bottini 2020 for reviews). Since there is no information about AM’s pre-morbid performance, it is not possible to reach further conclusions about the actual impact of the tumor. However, it is important to note that he still showed a typical distortion pattern.

In contrast to the stable cognitive and motor performance across sessions, we observed an overall trend indicating a reduction of the distortion related to the right hand representation. This was particularly evident for the hand width. In the literature, overestimation of the hand width and underestimation of the finger length have been associated with the size of the receptive fields and the cortical representation for these body parts (Longo and Haggard 2010, 2011, 2012a). In detail, the areas that occupy a larger surface in the cortex are, in turn, perceived as larger (Miller et al. 2016). Therefore, disruption or increase of the activity in these cortical areas may, in turn, affect the perceived size of the body. Increasing the size of a body part does enhance the activation of the somatosensory cortex (D’Amour and Harris 2017), in the same way that direct cortical activation of these areas through repetitive transcranial magnetic current stimulation (rTMS) increases the perceived size of the hand (Giurgola et al. 2019). We cannot comment on AM’s specific impact of tumor compression on the somatosensory cortex of the hand, but the width showing a more significant improvement after tumor resection suggests that modulation can be highly selective.

Improvement of body representation deficit after tumor resection has been occasionally reported previously. In particular, patients with meningiomas in the parietal–occipital areas that showed impairments in body-related tasks, such as left–right orientation, recovered their functions once the tumor was removed (Nikishina et al. 2016). Partial recovery was observed on a patient who showed a severe misoplegia toward her left leg, after the removal of a tumor in the right medial and temporoparietal lobes (Loetscher 2006). Also, the preservation of sensorimotor functions we observed in AM is in line with available data (Seitz et al. 1995). Still, it is of interest to note that the modulation of the performance was only evident in the perceptual task, but not in the motor task. Further studies could help elucidate if there is an association between better hand size representation and general motor performance due to bodybuilding.

It is important to note that the dimension where we found a change post-surgery was the width, but not the length. Indeed, variability in length perception seems less frequent, whereas width appears more susceptible to change (see Cocchini et al. 2018; Coelho et al. 2019; Longo 2022; Mora et al. 2021). One reason for this finding is that width is intrinsically related to more representational flexibility to accommodate growth (De Vignemont et al. 2005; Hashimoto and Iriki 2013). Moreover, width is the dimension that appears more linked to own body representation (Ganea and Longo 2017). For instance, length underestimation is found when judging the size of a rubber hand, but not width overestimation (Longo et al. 2015; Saulton et al. 2016). Instead, length underestimation appears to be more related to conceptual biases (Ambroziak et al. 2018; Longo 2015b; Longo et al. 2015; Margolis and Longo 2014), whereas width is a ‘pure’ perceptual distortion (i.e., spatial warping of the hand tissue) (Longo et al. 2015). For all these reasons, it is not surprising that it is the width dimension that is more susceptible to change.

We cannot exclude that some degree of familiarization may have played a role in the post-surgery performance; however, this seems unlikely since there was a gap of 12 months between testing sessions and no feedback was provided during the task, ensuring the patient was not aware of any possible distortion. Moreover, a recent study by Peviani and Bottini (2020) has shown that performance in the hand localization task is rather stable even after increased practice (number of trials).

Lastly, expertise in the use of hand, as in sign language interpreters (Allen et al. 2013; Mora et al. 2021; Penhune et al. 2003) or magicians (Cocchini et al. 2018), has been associated with better representation of hands. It is important to note that AM was a professional bodybuilder. We do not have information about pre-morbid (before the appearance of the tumor) body representation of AM, but we cannot exclude that he had a particularly refined pre-morbid representation of his hands. If this was the case, the pre-surgery distortion of the hand, in particular of the dorsum’s width, may have been attenuated by a pre-morbid proficiency. However, it could be that practice in bodybuilding does not influence the representation of the hands, as bodybuilding is not specifically associated with the use of hands and fingers, as in the case of magic and sign language interpreters. Indeed, body representation malleability is closely associated with functionality and the way the body is used (Caggiano et al. 2021; Caggiano and Cocchini 2020; Di Russo et al. 2006; Peviani et al. 2021; Romano et al. 2019). Instead, previous research has shown that the practice of bodybuilding increases the appearance of muscle dysmorphia and associated nutritional and drug disorders (Lantz et al. 2002).

Even though we did not find any impact on hand motor function before or after surgery, we did find an improvement in the way the hand was represented after tumor removal. Tumors are characterized by disrupting the activity of adjacent areas (Wunderlich et al. 1998) and some functions can be compensated through topographical reorganization of specific body parts (Ebeling et al. 1992). This may underlie important mechanisms of functional recovery. The results reported in this study support a direct involvement of sensorimotor areas in the implicit representation of the body size and suggests that limited damage of these brain areas can selectively affect different dimensions of body size.

## Data Availability

The data that support the findings of this study are available from the corresponding authors upon reasonable request.
